# Cancer of the Uterus

**Published:** 1899-11-25

**Authors:** 


[1] Lancet, Nov. 18.

### CANCER OF THE UTERUS.

Mr. F. Bowreman Jessett[1] records the results of 107 cases in which he has performed the operation of vaginal hysterectomy for carcinoma uteri during the last seven years. There can be no doubt that the results so far as they go are good. Whether they teach that the vaginal method is the best is another question. It is not impossible that recent investigations as to the tracks by which cancerous infection is spread may have the effect of turning surgeons back again towards the abdominal route, for the purpose of clearing out the possibly infected lymphatics, notwithstanding the proved facility with which the organ can be removed by way of the vagina. This is a question which may have to be reconsidered. In the meantime, however, it is well to note what has been done even by vaginal hysterectomy. Of the 107 cases 9 died from the operation. Of the 4 patients operated on in 1892, 1 is known to be well at the present time; of the 17 patients operated on in 1893 3 have been lost sight of, but they were well twelve months after the operation, and 5 were free from recurrence when last seen two and three years after the operation; of the 22 patients operated on in 1894, 7 were well when they were last seen, from nine to fifteen months after operation, and 6 others were well after two years; of the 16 patients operated on in 1895 10 were well when they were last seen, and 4 of these have been seen or reported on quite recently. Of the cases reported on more recently we need not give particulars, but we have quoted sufficient to show that, although recurrence takes place in a considerable number of cases, there remains a not inconsiderable proportion in which cure may fairly be said to have followed the operation, taking the word "cure" in the same sense as it is now commonly used in regard to cancer of the other organs. The most useful line of advance, as things stand at present, would seem to lie in the direction of earlier recognition of the disease and greater readiness to accept operation, not as being merely the only chance, but as the proper and legitimate course to recommend. In regard to this we will quote Mr. Bowreman Jessett, who says: "1. If on examination I found a patient at or past the menopause who was suffering from a discharge from the uterine cavity, which discharge was coloured and offensive, and in whose case the introduction of the sound caused bleeding, I should feel pretty certain that I had to deal with a case of carcinoma of the body of the uterus, and I should advise operation. Should any doubt, however, exist I would dilate the cervical canal and curette the cavity, and have the débris examined microscopically. If the report of the pathologist were unfavourable I should at once urge operative measures for the removal of the entire organ. 2. If the disease had commenced in the cervical canal or the external os, even if the walls of the vagina were somewhat invaded, so long as the uterus itself was not fixed or the broad ligament invaded, I should not hesitate to advise operation. Even in advanced cases so long as the uterus is movable I am convinced that much relief may be afforded and life may be prolonged by vaginal hysterectomy."

